# Minimally invasive medial femoral approach to total knee arthroplasty improves short-term outcomes compared to the standard medial parapatellar approach: a systematic review and meta-analysis

**DOI:** 10.1186/s13018-023-04136-2

**Published:** 2023-09-04

**Authors:** Xin Yang, Qing-hao Cheng, Yong-ze Yang, An-ren Zhang, Hua Fan, Hong-zhang Guo

**Affiliations:** 1 First Clinical Medical College of Gansu University of Chinese Medicine (Gansu Provincial Hospital), Lanzhou, China; 2 Gansu Provincial Hospital, 204 Donggang West Road, Chengguan District, Lanzhou, 730000 China

**Keywords:** Total knee arthroplasty, Medial parapatellar approach, Midvastus approach, Meta-analysis

## Abstract

**Objective:**

The aim of this study is to conduct a comprehensive evaluation of the effectiveness of the medial parapatellar approach via the vastus medialis obliquus muscle in comparison with the standard medial parapatellar approach for total knee arthroplasty, using a systematic approach.

**Methods:**

A computer search was conducted on PubMed, EMBASE, Medline, Cochrane libraries, and Web of Science databases to comprehensively collect randomized controlled studies on minimally invasive (MMV) approaches for knee arthroplasty, specifically the vastus and medial parapatellar (MP) approaches. Two authors independently screened the literature based on inclusion and exclusion criteria, evaluated the quality of the included studies using the Cochrane systematic review method, and performed a meta-analysis using RevMan 5.3 software.

**Results:**

A total of twelve randomized controlled studies were ultimately included, comprising 788 knees. The small incision medial femoral muscle approach (MMV) group consisted of 398 cases, while the traditional parapatellar approach (MP) group consisted of 390 cases. Data analysis showed that in the comparison of KSS, VAS, and ROM score at 3 months after surgery, MMV approach was superior to MP approach [MD = 2.89, 95%CI (0.33, 5.46),* P* = 0.03], [MD = − 0.22, 95%CI (− 0.36, − 0.09), *P* = 0.001], and [MD = 1.08, 95%CI (0.04, 2.12), *P* = 0.04]. However, there was no significant difference in the postoperative KSS, VAS, and ROM score between the MMV and MP approaches at 6 and 12 months after surgery. The operation time of the MMV group was longer than that of the MP group [MD = 8.98, 95%CI (4.64, 13.32), *P* < 0.0001], and the number of days of straight leg raising after surgery was shorter in the MMV group than in the MP group [MD = − 1.91, 95%CI (− 3.45, − 0.37),* P* = 0.01], with statistically significant differences. There was no significant difference in the lateral support band release rate [OR = 0.72, 95%CI (0.23, 2.28), *P* = 0.58], length of hospital stay [MD = 0.07, 95%CI (− 0.18, 0.31), *P* = 0.58], postoperative complications [MD = 0.62, 95%CI (0.33, 1.18), *P* = 0.15], and intraoperative blood loss [MD = 70.50, 95%CI (− 57.51, 198.72), *P* = 0.28].

**Conclusion:**

Most of the approaches have similar length of stay and incidence of complications compared to standard approaches. However, the minimally invasive midvastus approach has shown potential to improve short-term outcomes.

*Study registration*: PROSPERO registration number CRD42023410583.

**Supplementary Information:**

The online version contains supplementary material available at 10.1186/s13018-023-04136-2.

## Introduction

At present, total knee arthroplasty (TKA) has become the final clinical treatment for advanced knee osteoarthritis [1]. It is estimated that about 10% of men over 60 years old worldwide have osteoarthritis, and about 18% of women have osteoarthritis [2]. At present, the most commonly used approach for total knee arthroplasty is the medial parapatellar (MP). The length of the incision is generally about 15 cm. This long incision combined with the turnover of the patella can provide a good surgical field, but there is also a 5–30% chance of patellofemoral problems, such as postoperative patellar subluxation and aseptic necrosis of the patella, complications that affect the recovery of knee extensor function [3]. The advancement of science and technology has led to the widespread adoption of minimally invasive surgery (MIS) in clinical practice, as it offers the potential to enhance short-term recovery and minimize complications. Research has demonstrated that MIS surgery can result in reduced postoperative pain [4–6], faster restoration of quadriceps function [4, 5, 7], improved early-stage knee mobility [4–8], and greater cost-effectiveness compared to standard total knee arthroplasty [9].

Although minimally invasive total knee arthroplasty currently has a high success rate, opponents of the minimally invasive approach argue that due to the challenges in achieving adequate exposure, there is a risk of suboptimal prosthesis positioning and poor postoperative force alignment, which may compromise long-term efficacy. Despite ongoing debate, minimally invasive knee joint technology has experienced significant advancements, with various minimally invasive techniques for total knee replacement being proposed. There are four main small incision approaches for MIS-TKA: minimally invasive midvastus (MMV), mini-medial parapatellar (MMP), nondestructive approach to quadriceps (QS), and mini-subvastus approach (MSV) [10]. In MIS TKA, subvastus, midvastus, and quads-sparing approaches are the most commonly alternatives to standard parapatellar approach [11, 12]. Subvastus and quads-sparing approaches preserved the knee extensor mechanism and thus were regarded as more minimally invasive than the parapatellar approach. However, the small surgical field and the increasing operative difficulty limit the popularity of these two approaches [8, 13]. As a compromise of these approaches, mini-midvastus approach was introduced as it minimized the vascular and muscular disruption of knee and provided a relatively better operative exposure [14]. Therefore, mini-midvastus approach has probably been the most popular approach in MIS TKA [15].

There is still considerable debate regarding the effectiveness of the MMV and MP approaches. Some studies suggest that there is no significant difference between the two methods [16, 17], while others hold divergent views [18, 19]. However, there is no evidence-based research comparing the effectiveness of the MMV approach to the standard medial parapatellar (MP) approach. This study aims to conduct a systematic review and meta-analysis of randomized controlled trials (RCTs) in order to compare the postoperative efficacy of the MMV approach and the standard medial parapatellar approach (MP) for total knee arthroplasty. The goal is to provide surgeons with the best evidence-based medical evidence to aid in their selection of surgical methods.

## Materials and methods

### Protocol and registration

This study was conducted based on Preferred Reporting Items for Systematic Reviews and Meta-Analyses (PRISMA) [20]. The protocol for this review has been registered in PROSPERO (CRD42023410583).

### Literature search strategy and selection

Search PubMed, EMBASE, Medline, Cochrane library, and Web of Science databases were to collect the literature on randomized controlled trials of TKA using MMV and MP approaches from database establishment to March 23, 2023. The search method combines free words and subject words and is adjusted according to the different characteristics of each database. See Additional file 1 for specific search strategies.

### Inclusion and exclusion criteria

#### Study type

This study will focus on completely randomized controlled trials that compare the clinical effects of MMV and MP approaches for TKA.

#### Patient type

Gender, age, weight, and prosthesis type do not limit patients undergoing their first total knee arthroplasty.

#### Intervention

This study compared the clinical efficacy of the MMV approach and MP approach for total knee arthroplasty. The two groups were comparable in terms of number, age, and body mass index (BMI).

#### Outcome indicators

(1) Main indicators: knee joint score (KSS); visual analog score (VAS); knee joint functional score (KFS); range of motion (ROM); lateral retinacular release; and straight leg raising days (SLR) and (2) secondary indicators: time of operation; amount of blood loss during operation; time of hospitalization; and postoperative complications (including deep venous thrombosis and infection).

### Exclusion criteria

Semi-randomized controlled trials and retrospective studies should be excluded. Studies with inconsistent baselines between experimental and control groups, unscientific comparison and grouping methods, ambiguous trial data, and patients with severe deformities, infections, and revision should also be excluded. Exclude duplicate publications, case reports, reviews or expert opinions.

### Literature quality evaluation

#### Included in the literature review

Two researchers (Xin Yang and Qing-hao Cheng) strictly screened the literature materials retrieved according to the inclusion and exclusion criteria mentioned above, and extracted relevant data from each literature material, and then cross-compared them. In case of disagreement, it was resolved through discussion or consultation with a third party (Hong-zhang Guo).

#### Documentation quality evaluation

Two researchers (Xin Yang and Qing-hao Cheng) assessed the risk of bias in randomized controlled trials based on the Cochrane Collaborative tool [21]. The Cochrane Collaboration tool consists of seven main areas: random sequence generation, assignment hiding, experimental blinding, blinding of result evaluation, completeness of results, selective result reporting, and other sources of bias. Each area is classified as low, high, and unclear bias risk.

#### Statistical analysis

Use the Review Manager 5.3 software recommended by the Cochrane Collaboration Organization for statistical analysis. The two-class variables (postoperative complications) were expressed using odds ratio (OR), continuity variables (KSS, VAS, ROM, SLR, time of surgery, length of stay, and amount of bleeding) using mean difference (MD), and 95% confidence interval (CI). Test and analyze statistical heterogeneity: if *P* ≤ 0.1, *I*^2^ > 50%, it indicates that there is heterogeneity between the test results. The reasons for the heterogeneity should be analyzed and treated by sensitivity analysis. For studies that cannot eliminate statistical heterogeneity, a random effect model should be used for combined analysis. Conversely, a fixed effect model is used.

## Results

### Document screening process and results

**Fig. 1:**
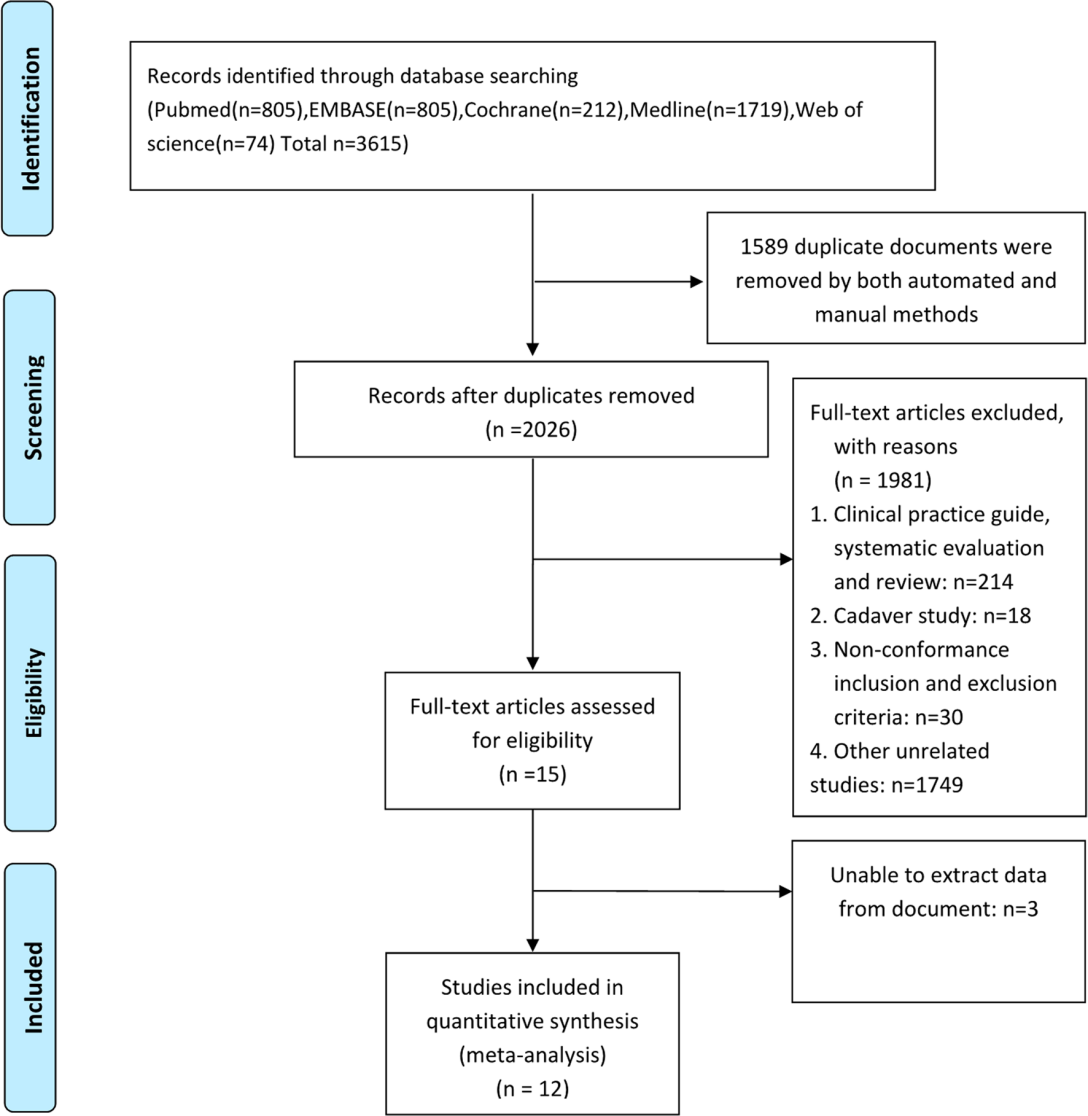
Preferred Reporting Items of Systematic Reviews and Meta-Analysis (PRISMA) flow diagram

A total of 3615 related documents were obtained in the preliminary screening, which were screened layer by layer and finally included in 12 studies, all of which were RCT. Detailed document screening process and results are shown in Fig. 1.

### Basic characteristics and bias risk assessment results incorporated into the study

**Fig. 2:**
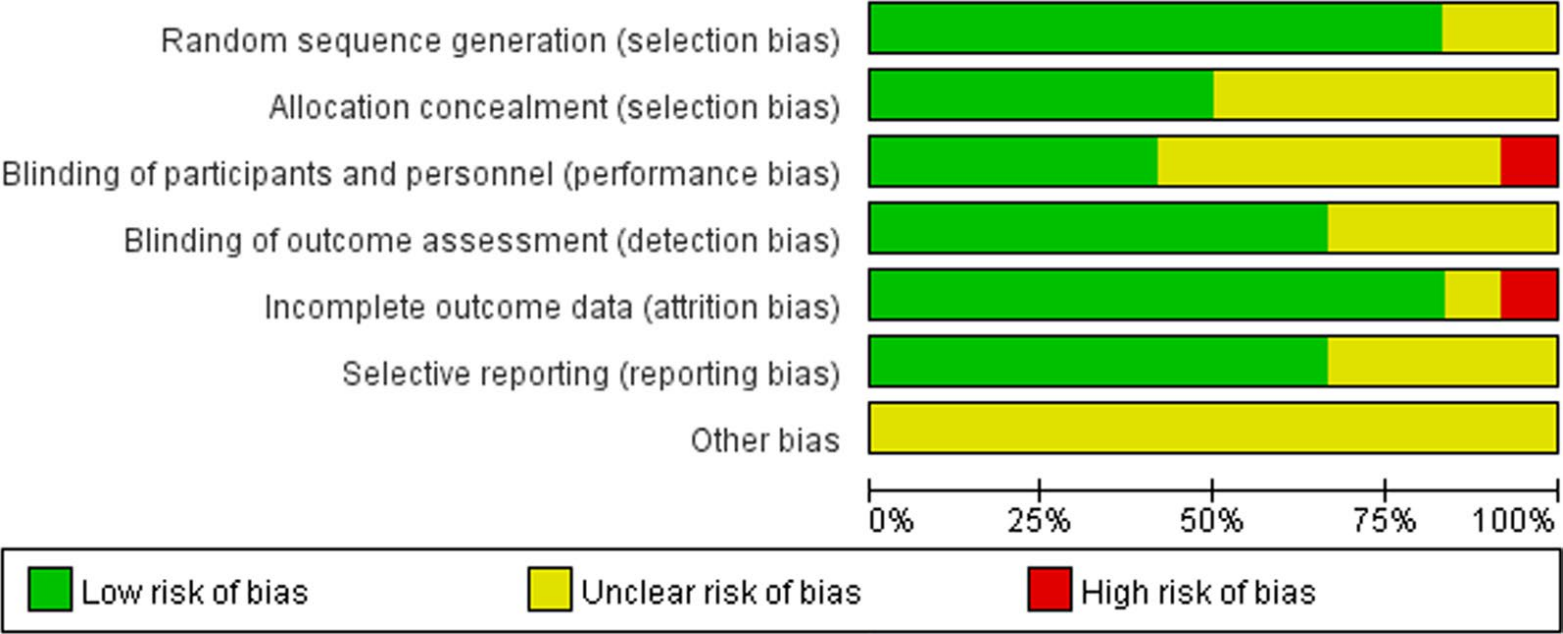
Bias risk distribution map for the literature

**Fig. 3:**
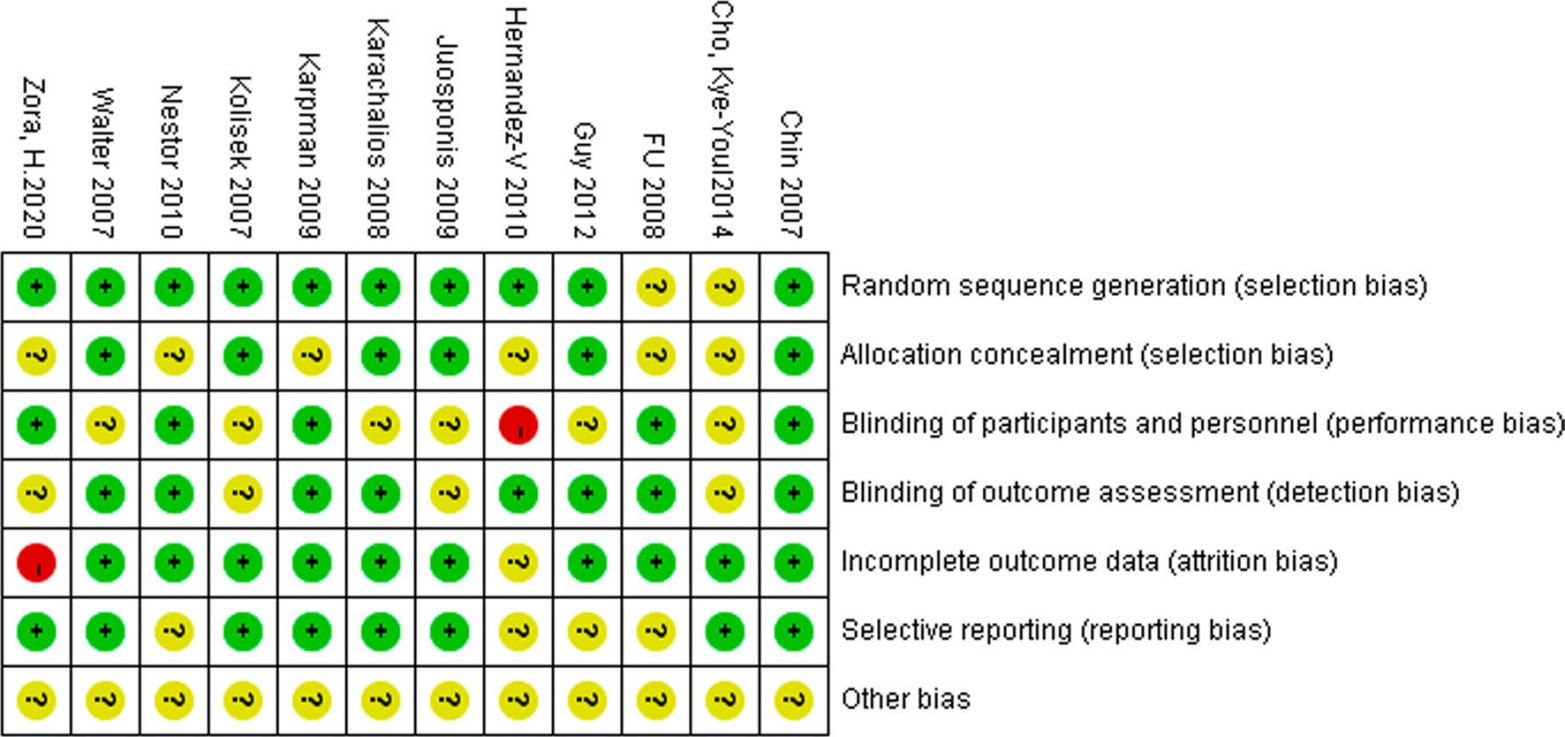
The literature bias risk summary map

The basic information included in the study is shown in Table 1. The results of the bias risk assessment included in the 12 studies are shown in Figs. 2 and 3.

**Table 1:** Basic information for incorporation into the literature

研究	年份	研究类型	关节例数 MMV/MP	年龄 MMV/MP	性别 (M/F)	结局指标		随访时间 (月)
Chin et al. [22]	2007	RCT	30/30	67.4/69	9/51	⑦⑧⑨⑩		3
Cho et al. [23]	2014	RCT	33/33	65.5 ± 5.1/67.0 ± 5.7	3/63	①③⑤⑦		12
Guy et al. [11]	2012	RCT	40/40	71.2/69.1	38/42	①③⑧⑨⑩		12
Hernandez-V et al. [24]	2010	RCT	26/36	70.8 ± 5.9/70.5 ± 6.9	11/51	③⑩		6
Juosponis et al. [25]	2009	RCT	35/35	72 ± 5.5/71.4 ± 5.04	10/60	①③⑤⑨⑩		3
Karachalios et al. [26]	2008	RCT	50/50	71.1/70.8	34/66	①⑤⑨⑩		36
Karpman et al. [27]	2009	RCT	20/19	74 ± 7.7/73 ± 5.1	16/23	②⑦⑧⑨⑩		6
Kolisek et al. [28]	2007	RCT	40/40	67/70	54/26	①⑤⑦⑧⑨⑩		3
Nestor et al. [29]	2010	RCT	27/27	66.7 ± 9.6	9/18	②③⑩		6
Walter et al. [30]	2007	RCT	36/19	71.5/66.6	17/38	②④⑥⑧		3
Fu et al. [31]	2008	RCT	34/34	67 (53–86)	7/27	③④⑥⑦⑨⑩		3
Zora, H. al.[32]	2020	RCT	27/27	65 ± 6.4/63.2 ± 6.3	4/50	⑤⑨⑧		3

F = Female; M = Male; ① = KSS; ② = VAS; ③ = ROM; ④ = Straight leg raise; ⑤ = KFS; ⑥ = Lateral retinacular release; ⑦ = Blood loss; ⑧ = Hospital stay; ⑨ = Operative time; and ⑩ = Complications

### Results

#### KSS of knee joint after operation

##### KSS 3 months after operation

**Fig. 4:**
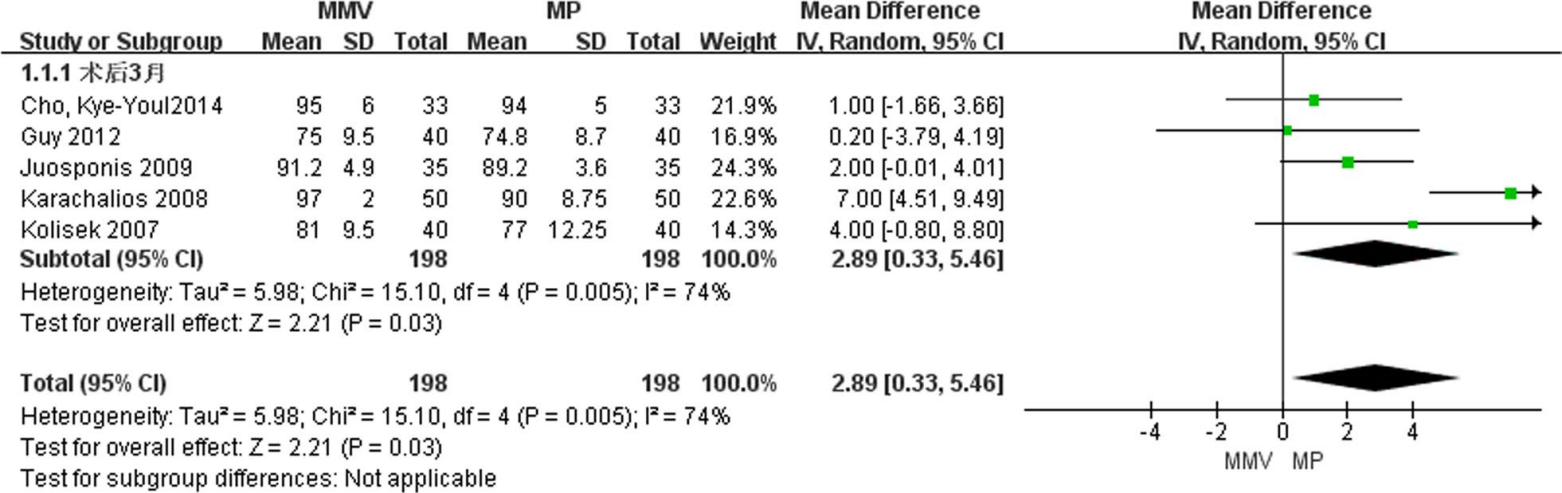
Forest map comparing KSS of knee joint 3 months after operation in two groups

A total of five articles [11, 23, 25, 26, 28] introduced KSS of knee joint at 3 months after operation, including 198 cases of MMV approach and 198 cases of MP approach. A random effect model was used to study the large heterogeneity between the two groups by the heterogeneity test (Chi^2^ = 15.10, *P* = 0.005, *I*^2^ = 74%). The results show that [MD = 2.98, 95%CI (0.33, 5.46), *P* = 0.03] MMV access KSS is better than traditional MP access (Fig. 4).

##### KSS 6 months after operation

**Fig. 5:**
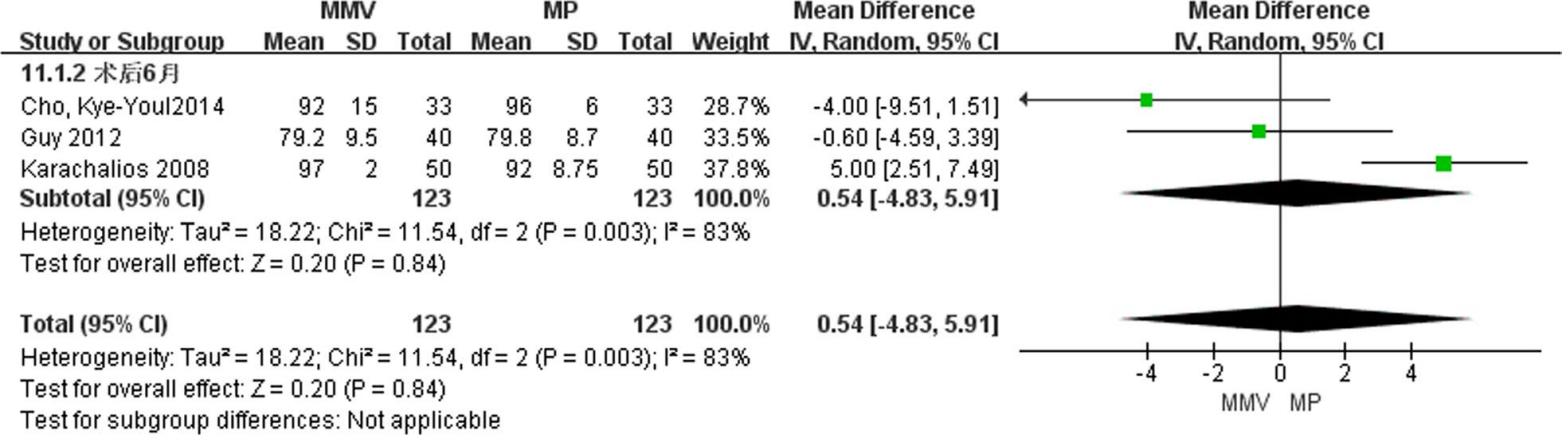
Forest map comparing KSS of knee joint 6 months after operation in two groups

Three studies [11, 23, 26] reported Knee Society Scores of knee joints at 6 months post-surgery, comprising of 123 medial parapatellar approaches and 123 medial pivot approaches. A random effects model was employed to investigate the substantial heterogeneity between the two groups (Chi^2^ = 11.54, *P* = 0.003, *I*^2^ = 83%). The findings indicated that there was no significant difference in KSS between the two approaches [MD = 0.54, 95%CI (− 4.83, 5.91), *P* = 0.84] (Fig. 5).

##### KSS 12 months after operation

**Fig. 6:**
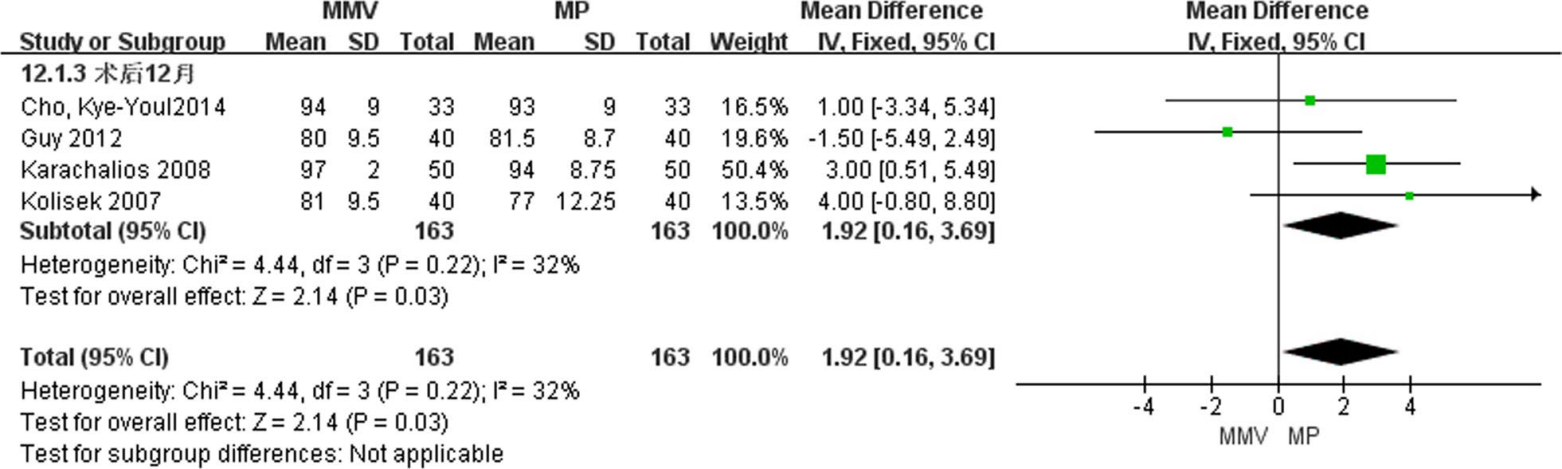
Forest map comparing KSS of knee joint between two groups at 12 months after operation

A total of four articles [11, 23, 26, 28] introduced KSS of knee joint at 12 months after operation, including 163 cases of MMV approach and 163 cases of MP approach. The small heterogeneity was studied by the heterogeneity test (Chi^2^ = 4.44, *P* = 0.22, *I*^2^ = 32%), and the fixed effect model was used. The results show that [MD = 1.92, 95%CI (0.16, 3.69), *P* = 0.03] MMV access KSS is better than traditional MP access (Fig. 6).

#### Postoperative knee pain

##### VAS of knee joint 3 months after operation

**Fig. 7:**
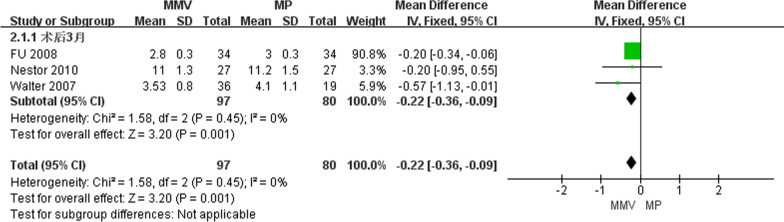
Forest map of knee joint pain at 3 months after operation in both groups

A total of three articles [29–31] introduced the knee joint VAS at 3 months after surgery, including 97 cases of MMV approach and 80 cases of MP approach. No heterogeneity was detected by heterogeneity test (Chi^2^ = 1.58, *P* = 0.45, *I*^2^ = 0%), and fixed effect model was used. The results show that [MD = − 0.22, 95%CI (− 0.36, − 0.09),* P* = 0.001] MMV access VAS is better than traditional MP access (Fig. 7).

##### VAS of knee joint 6 months after operation

A total of one article [27] introduced the knee joint VAS at 6 months after surgery, including 20 cases of MMV approach and 19 cases of MP approach. The results of systematic evaluation showed that [MD = − 1.40, 95%CI (− 2.82, 0.02), *P* = 0.05] there was no significant difference in VAS between the two approaches.

##### VAS of knee joint at 12 months after operation

A total of one article [29] introduced the knee joint VAS at 12 months after surgery, including 27 cases of MMV approach and 27 cases of MP approach. The results of systematic evaluation showed that [MD = − 0.20, 95%CI (− 0.95, 0.55),* P* = 0.60] there was no significant difference in VAS between the two approaches.

#### Functional score of knee joint after operation

##### Knee function score 3 months after operation

**Fig. 8:**
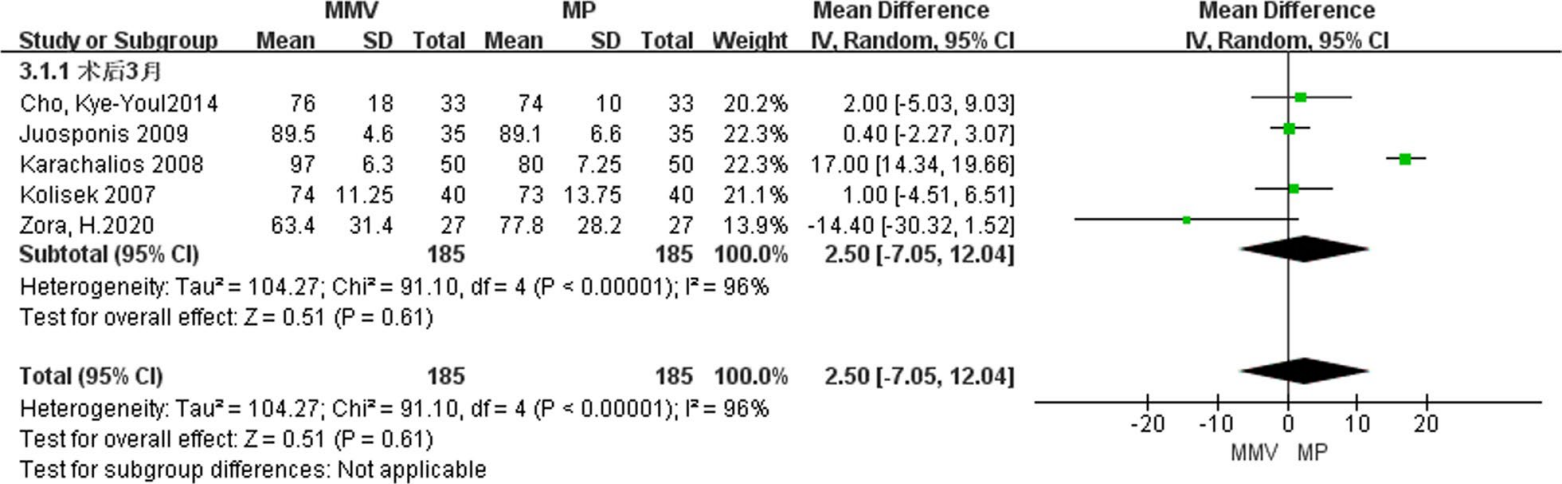
Forest map comparing knee function scores at 3 months after surgery in both groups

A total of five articles [23, 25, 26, 28, 32] introduced knee function scores at 3 months after surgery, including 185 cases of MMV approach and 185 cases of MP approach. A random effect model was used to study the large heterogeneity between the two groups (Chi^2^ = 91.10, *P* < 0.00001, *I*^2^ = 96%). The results suggest that [MD = 2.50, 95%CI (− 7.05, 12.04), *P* = 0.61] there was no significant difference in knee function scores between the two approaches (Fig. 8).

##### Knee function score 6 months after operation

**Fig. 9:**
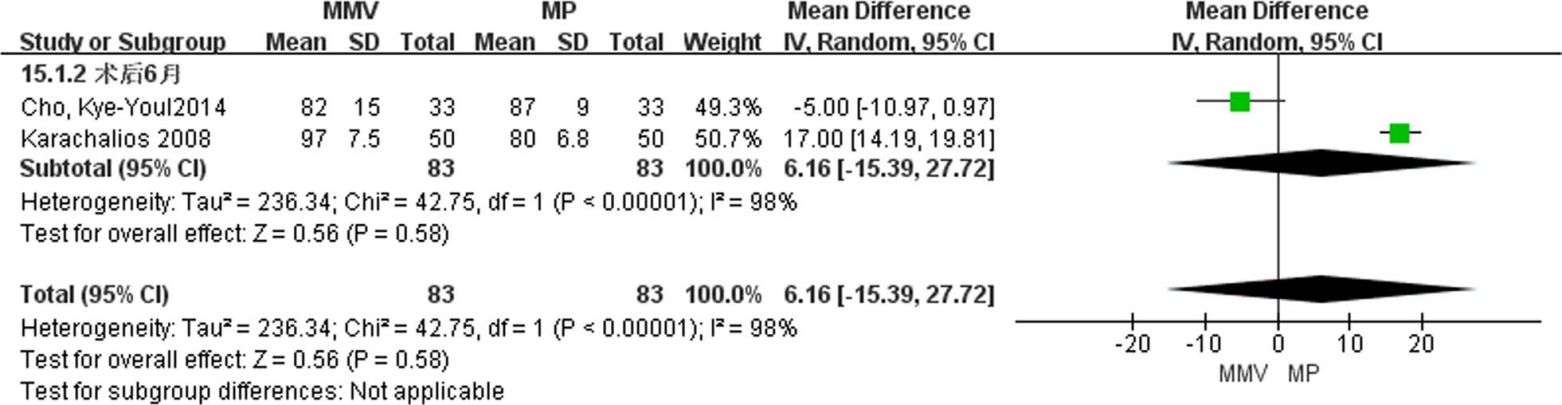
Forest map comparing knee function scores at 6 months after operation in both groups

A total of two articles [23, 26] described knee function scores at 6 months after surgery, including 83 cases of MMV approach and 83 cases of MP approach. A random effect model was used to study the large heterogeneity between the two groups (Chi^2^ = 42.75, *P* < 0.00001, *I*^2^ = 98%). The results suggest that [MD = 6.16, 95%CI (− 15.39, 27.72), *P* = 0.58] there was no significant difference in knee function scores between the two approaches (Fig. 9).

##### Knee function score at 12 months after operation

**Fig. 10:**
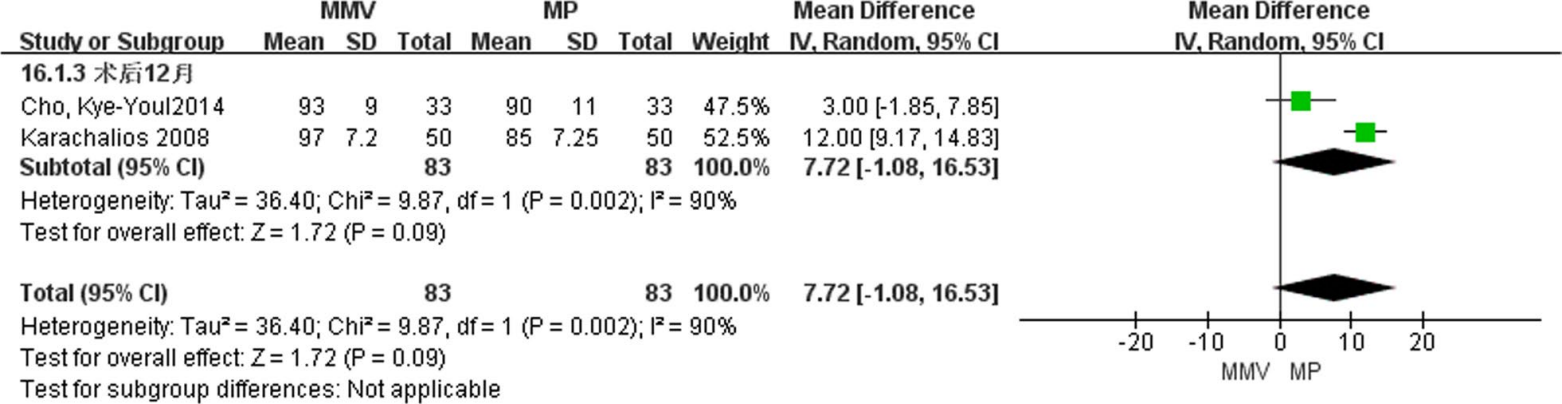
Forest maps comparing knee function scores at 12 months after surgery in both groups

A total of two articles [23, 26] introduced knee function scores at 12 months after surgery, including 83 cases of MMV approach and 83 cases of MP approach. A random effect model was used to study the large heterogeneity between the two groups by the heterogeneity test (Chi^2^ = 9.87,* P* = 0.002, *I*^2^ = 90%). The results suggest that [MD = 7.72, 95%CI (− 1.08, 16.53), *P* = 0.09] there was no significant difference in knee function scores between the two approaches (Fig. 10).

#### Mobility of knee joint buckling after operation

##### Knee joint flexural activity 3 months after operation

**Fig. 11:**
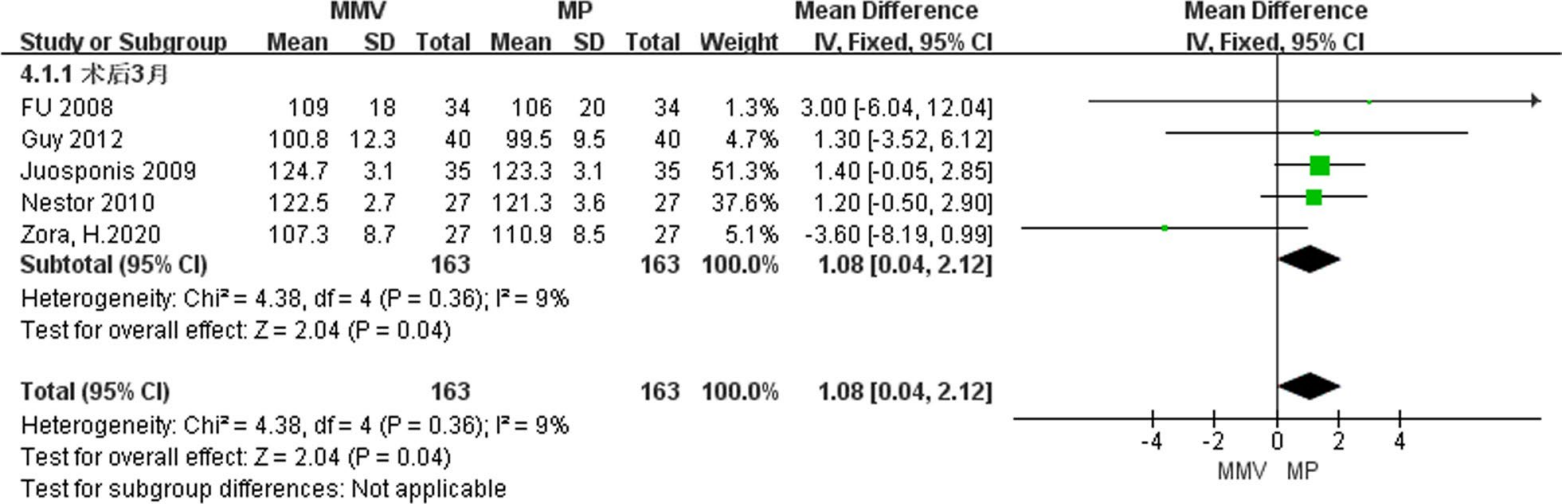
Forest map of knee joint flexural activity at 3 months after operation in both groups**s**

A total of five articles [11, 25, 29, 31, 32] described the degree of knee flexural activity at 3 months after surgery, including 163 cases of MMV approach and 163 cases of MP approach. The heterogeneity test (Chi^2^ = 4.38, *P* = 0.36,* I*^2^ = 9%) was used to study the small heterogeneity between the two groups. The results suggest that [MD = 1.08, 95%CI (0.04, 2.12),* P* = 0.04] MMV approach is superior to conventional MP approach in knee flexural activity (Fig. 11).

##### Knee joint flexural activity 6 months after operation

**Fig. 12:**
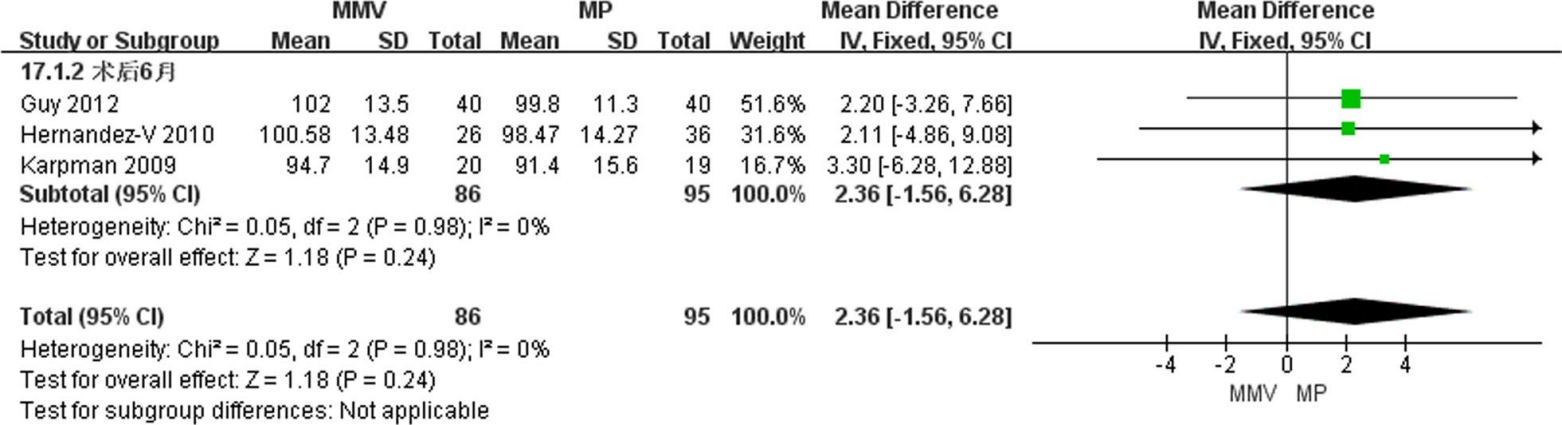
Forest map of knee flexural activity at 6 months after surgery in both groups

A total of three articles [11, 24, 27] reported on the degree of knee flexion activity at 6 months post-surgery, comprising 86 cases of MMV approach and 95 cases of MP approach. The heterogeneity test (Chi^2^ = 0.05, *P* = 0.98, *I*^*2*^ = 0%) indicated no heterogeneity, and a fixed effect model was employed. The results suggest that there is no significant difference in knee joint flexion activity between the two approaches [MD = 2.36, 95%CI (− 1.56, 6.28), *P* = 0.24] (Fig. 12).

##### Knee joint flexural activity at 12 months after operation

**Fig. 13:**
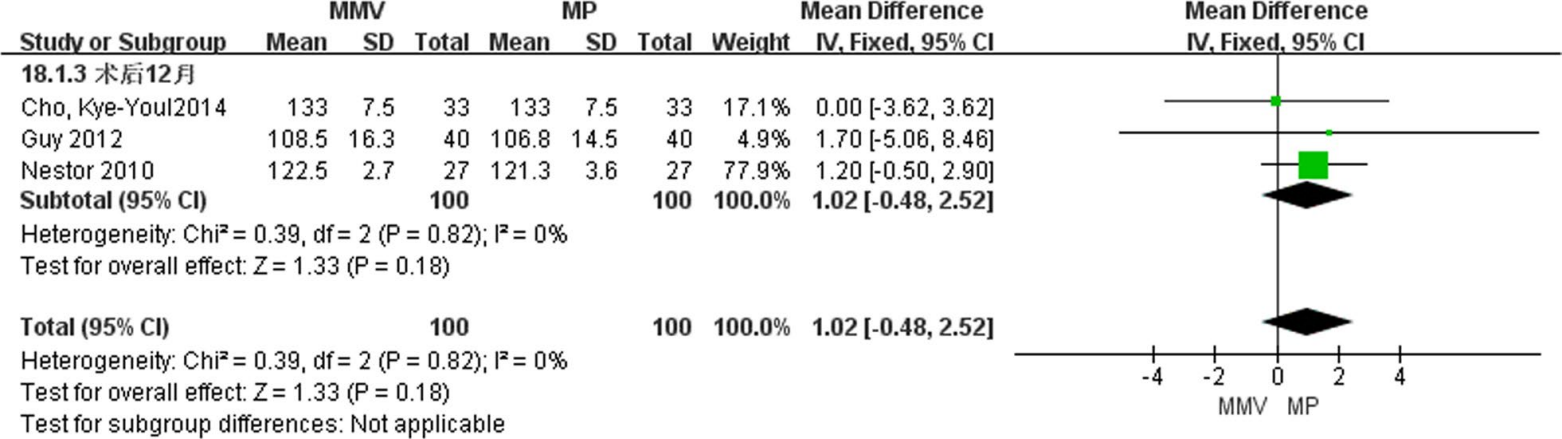
Forest map of knee flexural activity at 12 months after surgery in both groups

A total of three articles [11, 23, 29] described the degree of knee flexural activity at 12 months after surgery, including 100 cases of MMV approach and 100 cases of MP approach. The heterogeneity test (Chi^2^ = 0.39, *P* = 0.82, *I*^2^ = 0%) showed that there was no heterogeneity between the studies, and the fixed effect model was used. The results suggest that [MD = 1.02, 95%CI (− 0.48, 2.52), *P* = 0.18] there is no significant difference in knee joint flexural activity between the two approaches (Fig. 13).

#### Days of straight leg raise after operation

**Fig. 14:**
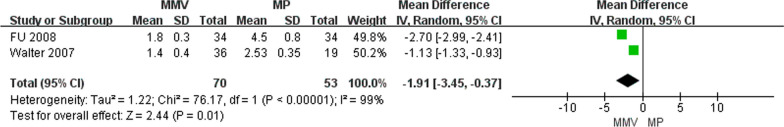
Forest map comparing days of straight leg elevation between the two groups

Two articles [30, 31] reported on the duration of postoperative straight leg raise, with 70 cases using the MMV approach and 53 cases using the MP approach. A heterogeneity test showed significant differences between the two studies (Chi^2^ = 76.17, *P* < 0.00001, *I*^2^ = 99%), and a random effects model was used for analysis. The results indicate that the MMV approach is superior to the MP approach in improving the duration of postoperative straight leg raise [MD = − 1.91, 95%CI (− 3.45, − 0.37), *P* = 0.01] (Fig. 14).

#### External support belt release rate

**Fig. 15:**
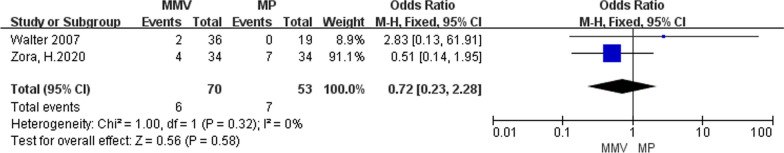
Forest map comparison of loose number of outboard support belts in two groups

Two articles [30, 32] have been identified that describe the rate of release of the lateral support band, comprising 70 MMV approaches and 53 MP approaches. The heterogeneity test revealed no significant heterogeneity between the two studies (Chi^2^ = 1.00, *P* = 0.32, *I*^2^ = 0%), and therefore, a fixed effect model was employed for analysis. The findings indicate that there was no significant difference between the two groups in terms of improving the release rate of the lateral support bands [OR = 0.72, 95%CI (0.23, 2.28),* P* = 0.58]. Efforts were made to ensure that the language used in this text is formal and academic (Fig. 15).

#### Operation time

**Fig. 16:**
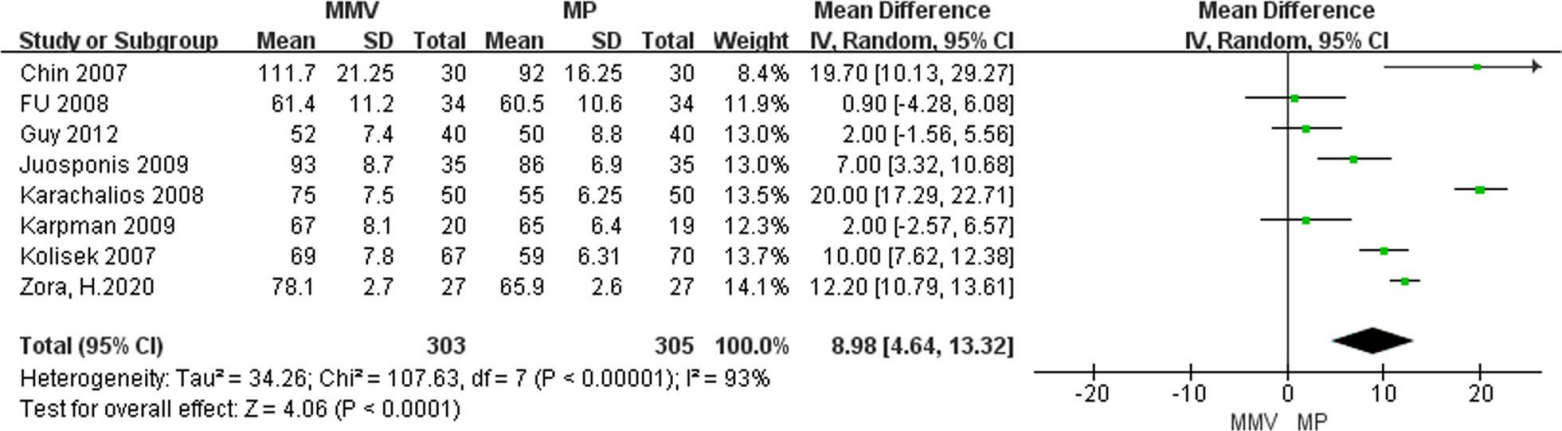
Forest map of two groups of surgical time comparisons

A total of eight articles [11, 22, 25–28, 31, 32] described the operation time, including 303 cases of MMV approach and 305 cases of MP approach. The results of meta-analysis showed that the heterogeneity between the studies was larger (Chi^2^ = 107.63, *P* < 0.00001, *I*^2^ = 93%), and the random effect model was used for analysis. The results suggest that the MMV approach group had longer surgical times than the traditional MP approach group [MD = 8.98, 95%CI (4.64, 13.32, *P* < 0.0001] (Fig. 16).

#### Days of hospitalization

**Fig. 17:**
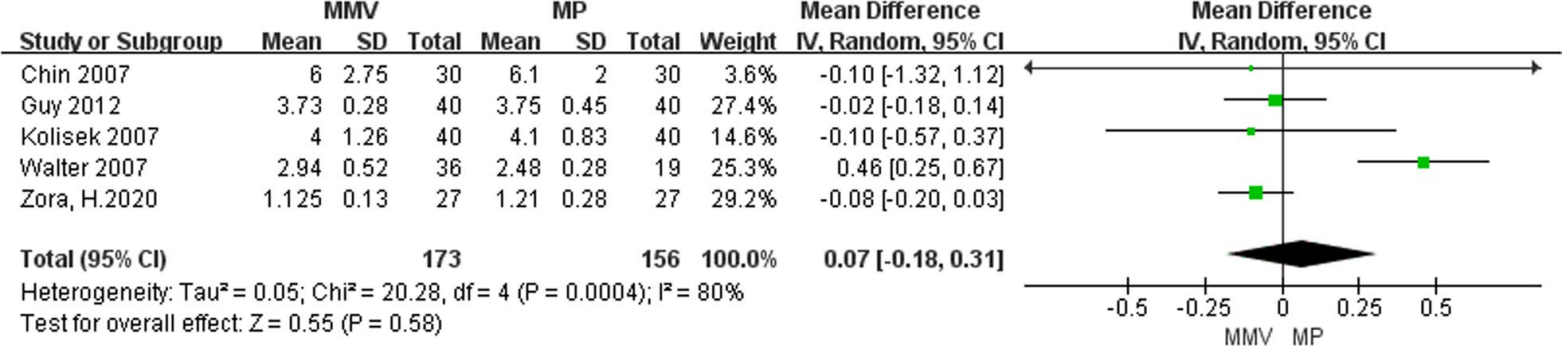
Forest map for comparison of hospital days in two groups

A total of five articles [11, 22, 28, 30, 32] described the number of days hospitalized, of which 173 were MMV access and 156 were MP access. The results of meta-analysis indicate that the heterogeneity between the studies is larger (Chi^2^ = 20.28, *P* = 0.0004,* I*^2^ = 80%), and the random effect model is adopted for analysis. The results suggest that there was no statistical difference in length of stay between the two groups [MD = 0.07, 95%CI (− 0.18, 0.31, *P* = 0.58] (Fig. 17).

#### Postoperative complications

**Fig. 18:**
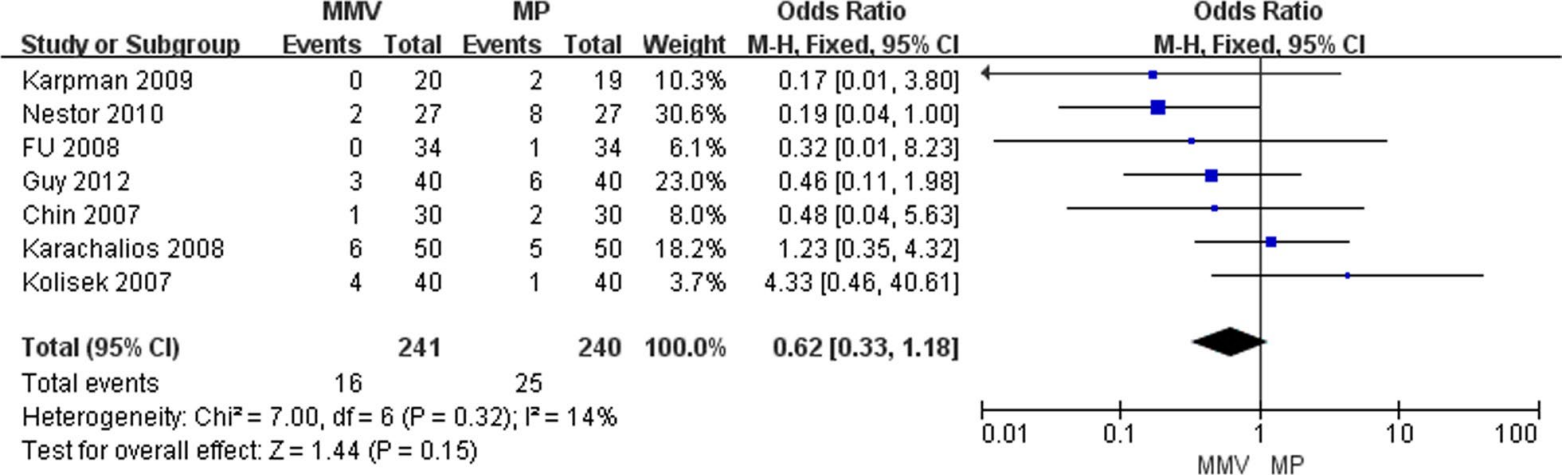
Forest map comparing postoperative complications between the two groups

The postoperative complications were described in seven articles [11, 22, 26–29, 31], including 241 cases of MMV approach and 240 cases of MP approach. The results of meta-analysis showed that the heterogeneity between the studies was small (Chi^2^ = 7.00, *P* = 0.32, *I*^2^ = 14%), and the fixed effect model was used for analysis. The results suggest that there was no significant difference in postoperative complications between MMV and standard MP approaches [OR = 0.62, 95%CI (0.33, 1.18, *P* = 0.15] (Fig. 18).

#### Amount of blood loss during operation

**Fig. 19:**
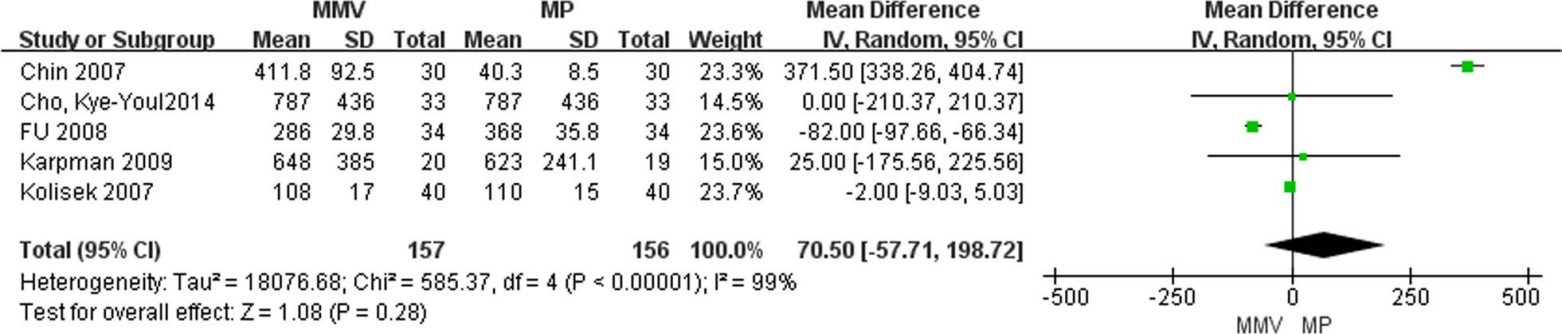
Forest map of blood loss in two groups during operation

A total of five articles [22, 23, 27, 28, 31] reported on the amount of blood loss during surgery, comprising 157 cases of MMV approach and 156 cases of MP approach. The meta-analysis results revealed significant heterogeneity between the studies (Chi^2^ = 585.37, *P* < 0.00001,* I*^2^ = 99%), necessitating the use of a random effects model for analysis. The findings indicate that there is no statistically significant difference in the amount of blood loss during surgery between the MMV approach and the standard MP approach [MD = 70.50, 95%CI (− 57.51, 198.72), *P* = 0.28] (Fig. 19).

### Publishing bias analysis

**Fig. 20:**
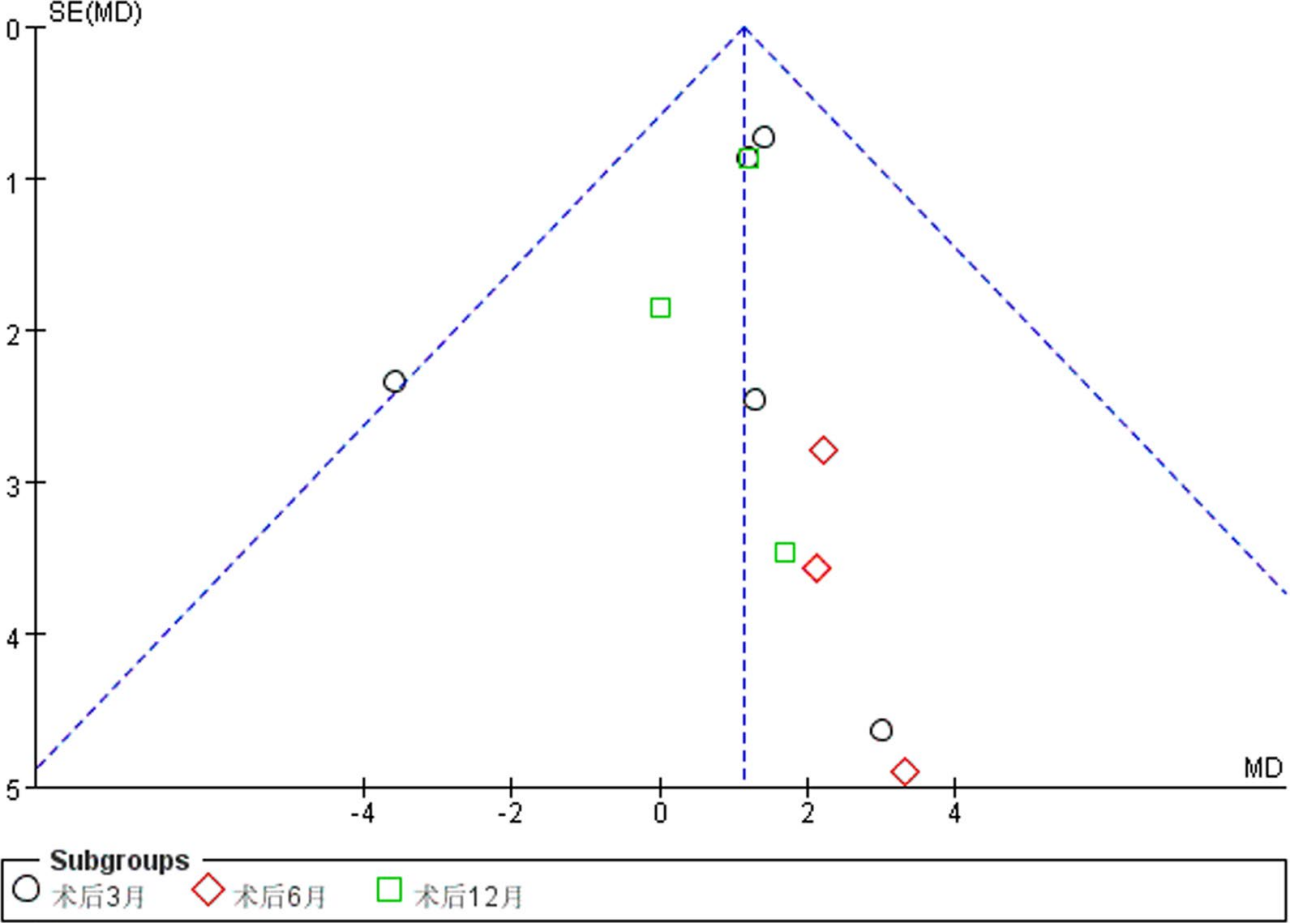
Released biased funnel chart of postoperative knee joint activity

The funnel chart of postoperative knee flexural activity shows that most of the data are concentrated in the middle and lower part, and the distribution is relatively symmetric. The proportion of the top part is small. Although there is a small amount of bias, the overall result is acceptable (Fig. 20).

## Discussion

Total knee arthroplasty (TKA) is an immensely beneficial and effective method for treating severe knee joint diseases. By reconstructing knee joints that conform to biomechanical characteristics and have standard valgus angles, TKA is primarily used to alleviate pain, restore anatomy and location, and improve daily functions [33].

The main difference between the MMV and MP approaches is that the quadriceps tendon and patellar ligament are torn in the MP approach. MP approach has a good surgical field of view, but due to the presence of insufficient exposure of the posterior joint structure, and easy to cause saphenous nerve infrapatellar branch injury, patellar dislocation, ischemic fracture, and other complications and affect the recovery of knee extensor function and other deficiencies [3]. Although MMV can keep the knee extension device intact, it also has the disadvantages of poor exposure during surgery, poor stability of the femoral joint, and poor functional recovery of the medial femoral muscle after operation [34]. Therefore, there is still widespread controversy over the clinical efficacy and postoperative complications of the two surgical approaches. There is no unified statement on what kind of surgery is better and can improve the quality of life of patients.

The KSS provides a comprehensive evaluation of knee pain, mobility, and stability in the anteroposterior, internal, and external directions. It also considers the impact of flexion contracture, extension lag, and adverse effects on alignment. The ROM score assesses knee range of motion, anatomical symmetry, repeatability of activity, and quality of activity. The VAS offers a straightforward assessment of patient pain. The results of this study suggest that MMV approaches are superior to MP approaches in early (postoperative March) KSS, VAS, and ROM score. However, there was no significant difference in KSS, VAS, and ROM score between the two groups in the middle and late stage (postoperative June and December). This suggests that the MMV technique provides superior joint mobility and pain perception in early-stage patients. In contrast with the MP approach, the MMV method eliminates the requirement for splitting the quadriceps tendon and patellar ligament. This not only results in a smaller surgical incision, but also reduces soft tissue damage and postoperative pain. As a result of the postoperative rehabilitation process, the patient's knee joint almost completely recovers, leading to a gradual reduction in differences between the two groups in various aspects. At 6 months and beyond, the patients in both groups exhibit similar KSS, VAS, and ROM scores. In a randomized controlled trial of 68 bilateral TKA reported by Fu et al. [31], significant reductions in VAS were also observed in the MMV group at week 1. For the KSS, there was no significant difference between MMV approach and MP approach 6 months after operation and 1 year after operation. This is consistent with several studies reported recently [17, 35]. Zhang [36] 45 MMV approaches were compared with 44 MP approaches, and there were no significant statistical differences in KSS at 6 weeks, 3 months, and 6 months of follow-up. Guy [11] eighty patients were randomized, and no statistically significant difference in KSS was found between the two groups at any point in time of follow-up.

The most crucial concern for clinicians following knee arthroplasty is the recovery of knee function, with the muscle strength of the quadriceps being the most reliable indicator of knee joint recovery [37]. In this study, we utilized the number of days of straight leg elevation post-surgery as the primary metric to evaluate recovery. The results demonstrated that the quadriceps recuperation was superior with the MMV technique in comparison with the MP method. To ensure the reliability and stability of the findings, we also included the relaxation rate of the lateral support band as a research indicator. The results revealed that the relaxation rate of the lateral support band in the MMV group did not differ significantly from that in the MP group. Documents show that [7] MMV approach requires release of soft tissue and lateral collateral ligaments in order to ensure early postoperation prosthesis stability and soft tissue balance due to small incision. The loosening of the lateral support belt is due to the dislocation of the patella and/or the inclination of the patella found during the operation, which reduces the tendency of the dislocation of the external patella by loosening the lateral support of the, improves the cheekbone force line and the trajectory of the patella, but can also lead to complications such as damage to the blood supply of the patella, necrosis of the patella, and poor healing of the soft tissue [38]. However, there is a body of the literature indicating that the medial patellofemoral ligament (MP) group also experienced relaxation of the lateral collateral ligament. Alcelik et al. [39] suggested that the medial meniscus vertical (MMV) suture has a lower relaxation rate than the MP lateral support band, but our study does not support this claim. Our analysis excluded non-randomized controlled trials and studies that did not provide raw data with ± SD to avoid data conversion errors.

The results showed that the operation time of MMV group was longer than that of MP group, and the difference was statistically significant. Karpman et al. [27] believe that MMV procedures are more difficult and time-consuming to expose surgical vision. Studies by Chin et al. [22] and Juosponis et al. [25] also show that MMV surgery requires more steps to strive to establish the most intuitive and optimized surgical window to ensure the identification of bone markers during surgery, the placement of surgical aids, and the safety and accuracy of prosthesis implantation, which are directly related to time-consuming.

The findings of this study indicate that there is no significant difference in postoperative complications between the MMV approach and the MP approach. However, Chin et al. [22] have reported that the MMV surgery demands higher technical and empirical skills from the surgeon as compared to MP surgery. Moreover, there are more operations performed in the joint cavity during MMV surgery, resulting in greater surgical damage and higher incidence of postoperative complications. Additionally, the use of lateral cutting fixtures and the accuracy of bone cutting operations are more challenging to achieve with MMV alone, which may affect component alignment or notching, leading to other complications. On the other hand, Nestor et al. [29] have demonstrated that MP surgery increases the likelihood of loose fixation, poor tibial rotation alignment, and unstable knee flexion. These findings are quite different from the results of this study and thus require further research to confirm the conclusion.

## Advantages and limitations of research

The inclusion criteria for this meta-analysis are strictly limited, with all semi-randomized controlled trials and non-randomized controlled trials being excluded to ensure more reliable results. Studies related to non-minimally invasive medial femoral muscle approaches were also excluded to minimize bias caused by different surgical methods, allowing for a focus on comparing the efficacy of the MMV approach and MP approach. However, this article still has some limitations. Like many other meta-analyses, the quality of the literature included is uneven, with a high risk of bias. Although 12 randomized controlled studies were included, there were few related major observations, making it difficult to evaluate publication bias through funnel plots. Additionally, there were differences in specific operating principles and management methods, including surgical techniques, the use of drainage or tourniquet applications, DVT precautions, and different prosthesis types, which may lead to related biases. Since the original studies did not include the outcome indicator of lower limb alignment, a comparative study of postoperative lower limb alignment could not be conducted. This indicator should be included in the future studies. Some included studies did not specify the specific methods of random allocation and hidden grouping, which may increase the risk of implementation bias and selection bias. Furthermore, there was no investigation of the long-term efficacy of the study, and there is a lack of long-term clinical evidence. Therefore, the results should be carefully considered.

## Conclusion

In summary, minimally invasive total knee arthroplasty is technically more difficult, less visualized, and has an inherent learning curve. Most approaches have similar length of stay and incidence of complications compared to standard approaches, but MMV approach can improve short-term outcomes.

### Supplementary Information

**Additional file 1:** Retrieval Policy.

## Data Availability

All data generated or analyzed during this study are included in this published article [and its Additional file 1].

## Abbreviations

MSH: Medical Subject Headings
RCT: Randomized controlled trials
WD: Weighted differences
OR: Odds ratio
95%CI: 95% confidence interval
NOS: The Newcastle–Ottawa Quality Assessment Scale
