# Supplementary material for: Minimally invasive medial femoral approach to total knee arthroplasty improves short-term outcomes compared to the standard medial parapatellar approach: a systematic review and meta-analysis
Source: J Orthop Surg Res. 2023 Sep 4;18:657. doi: 10.1186/s13018-023-04136-2 (PMC10478389; doi:10.1186/s13018-023-04136-2)
Supplement: Supplementary file 1 — Additional file 1: Retrieval Policy. [file 13018_2023_4136_MOESM1_ESM.docx]

**Retrieval Strategy**

**Pubmed：N=805**

((Knee arthroplasty[Text Word]) OR (Knee replacement[Text Word])) AND ((((mini-midvastus[Text Word]) OR (mini-medial parapatellar[Text Word])) OR (limited medial parapatellar[Text Word])) OR (minimally invasive[Text Word]))

**Cochrane：N=212**

#1 (Knee arthroplasty):ti,ab,kw OR ("knee replacement"):ti,ab,kw

#2 (min-midvastus)ti ab .kw OR (mini-medial parapatellar)ti.ab kw OR (limited medial parapatella)ti ab kw OR (minimaly invasive)fi ab kw

#3 #1 and #2

**Embase：N=805**

#1. 'knee arthroplasty':ti,ab,kw OR 'knee 46,620 26 Mar 2023

replacement':ti,ab,kw

#2. 'mini-midvastus':ti,ab,kw OR 'mini-medial 133,256 26 Mar 2023

parapatellar':ti,ab,kw OR 'limited medial

parapatellar':ti,ab,kw OR 'minimally

invasive':ti,ab,kw

#3. #1 AND #2 805 26 Mar 2023

**Medline：N=1719**

S1 TX mini-midvastus OR TX mini-medial parapatellar OR TX limited medial parapatellar OR TX minimally invasive

S2 TX knee arthroplasty or knee replacement or total knee replacement

S3 S1 and S2

**Web of science： N=74**

#1 Knee arthroplasty (主题) or Knee replacement (主题)

#2 mini-midvastus (主题) or mini-medial parapatellar (主题) or limited medial parapatellar (主题) or minimally invasive (主题)

#3 #1 and #2
